# 2-(9-Anthrylmethyl­ideneamino)-4-methyl­phenol

**DOI:** 10.1107/S1600536810016715

**Published:** 2010-05-15

**Authors:** Andrés Villalpando, Frank R. Fronczek, Ralph Isovitsch

**Affiliations:** a Department of Chemistry, Whittier College, 13406 Philadelphia Street, Whittier, CA 90608, USA; b Department of Chemistry, Louisiana State University, Baton Rouge, LA 70803, USA

## Abstract

The title compound, C_22_H_17_NO, is a novel Schiff base synthesized *via* a condensation reaction between 9-anthracenecarboxaldehyde and 2-amino-*p*-cresol. The asymmetric unit contains two independent mol­ecules that are joined by an O—H⋯OH hydrogen bond. An intra­molecular O—H⋯N hydrogen bond occurs in each mol­ecule. π-stacking about inversion centers was observed between adjacent phenol rings [centroid–centroid distance = 3.850 (2) Å] and adjacent anthracene rings [centroid–centroid distance = 3.834 (2) Å]. The C—N=C—C torsion angles between the phenol and anthracene rings are close to 180° with values of 174.06 (15) and 179.85 (14)°.

## Related literature

For related structures, see: De *et al.* (2008 ▶); Ünver *et al.* (2009 ▶). For bond-length data, see: Allen *et al.* (1987 ▶). For background to the use of luminescent metal complexes formed by Schiff bases in light emitting diode construction and solar energy collection, see: Liao *et al.* (2009 ▶); Mak *et al.* (2009 ▶).
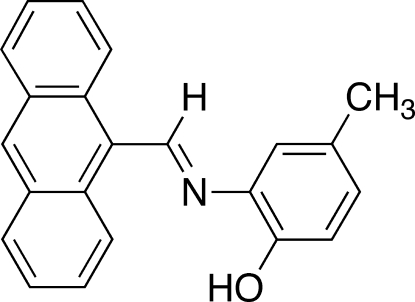


## Experimental

### 

#### Crystal data

C_22_H_17_NO
*M* *_r_* = 311.37
Triclinic, 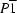

*a* = 8.6037 (15) Å
*b* = 12.839 (3) Å
*c* = 15.015 (3) Å
α = 94.508 (9)°
β = 97.164 (11)°
γ = 106.490 (11)°
*V* = 1566.6 (6) Å^3^
*Z* = 4
Mo *K*α radiation
μ = 0.08 mm^−1^
*T* = 90 K
0.37 × 0.15 × 0.05 mm

#### Data collection

Nonius KappaCCD diffractometer with Oxford Cryostream
35942 measured reflections
7462 independent reflections
4454 reflections with *I* > 2σ(*I*)
*R*_int_ = 0.057

#### Refinement

*R*[*F* ^2^ > 2σ(*F* ^2^)] = 0.054
*wR*(*F* ^2^) = 0.127
*S* = 1.02
7462 reflections
442 parameters
H atoms treated by a mixture of independent and constrained refinement
Δρ_max_ = 0.30 e Å^−3^
Δρ_min_ = −0.28 e Å^−3^

### 

Data collection: *COLLECT* (Nonius, 2000 ▶); cell refinement: *DENZO* and *SCALEPACK* (Otwinowski & Minor, 1997 ▶); data reduction: *DENZO* and *SCALEPACK*; program(s) used to solve structure: *SIR97* (Altomare *et al.*, 1999 ▶); program(s) used to refine structure: *SHELXL97* (Sheldrick, 2008 ▶); molecular graphics: *ORTEP-3 for Windows* (Farrugia, 1997 ▶); software used to prepare material for publication: *SHELXL97*.

## Supplementary Material

Crystal structure: contains datablocks I, global. DOI: 10.1107/S1600536810016715/zq2039sup1.cif

Structure factors: contains datablocks I. DOI: 10.1107/S1600536810016715/zq2039Isup2.hkl

Additional supplementary materials:  crystallographic information; 3D view; checkCIF report

## Figures and Tables

**Table 1:** Hydrogen-bond geometry (Å, °)

*D*—H⋯*A*	*D*—H	H⋯*A*	*D*⋯*A*	*D*—H⋯*A*
O1—H10*H*⋯N1	0.82 (2)	2.27 (2)	2.754 (2)	118.0 (17)
O2—H20*H*⋯O1	0.86 (2)	2.11 (2)	2.8602 (18)	144.9 (18)
O2—H20*H*⋯N2	0.86 (2)	2.17 (2)	2.695 (2)	119.1 (17)

**Fig. 1.**
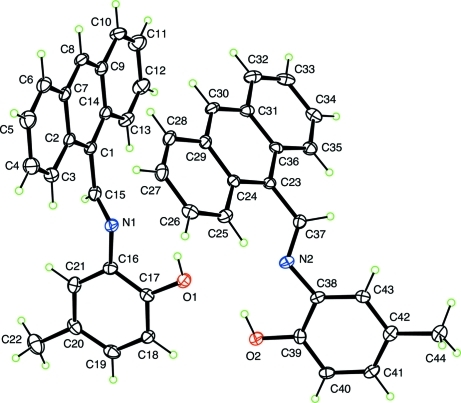
The asymmetric unit with ellipsoids at the 50% probability level and H atoms having arbitrary radius.

## supplementary crystallographic information

### Comment

Schiff bases can form luminescent metal complexes that are used in research areas that range from light emitting diode construction to solar energy collection (Liao *et al.*, 2009; Mak *et al.*, 2009). Our research explores the synthesis and photophysics of novel anthracenyl Schiff bases and their metal complexes toward the goal of utilizing them in the preparation of light emitting diodes.

The structure of the title compound is shown in Figure 1. The asymmetric unit is comprised of two independent molecules of the title compound joined together by a hydrogen bond of length 2.8602 (18) Å, which is formed from the interaction of the OH groups on the phenol rings. π-stacking about inversion centers was observed between adjacent phenol rings with a centroid-centroid distance of 3.850 Å and between adjacent anthracene rings with a centroid-centroid distance of 3.834 Å.

There is slight variation in the bond lengths and angles of the two independent molecules. The central C—N double bond, C15—N1, has a bond length of 1.280 (2) Å. This bond length is close to the literature value of 1.279 Å for a C(sp^2^)═N(sp^2^) bond (Allen *et al.*, 1987). The C—C bond, C1—C15 and C23—C37, that connects the anthracene to the central C—N double bond has bond lengths of 1.477 (2) and 1.470 (2) Å, respectively. The C—N bond, N1—C16 and N2—C38, that connects the phenyl ring to the central C—N double bond has bond lengths of 1.419 (2) and 1.414 (2) Å, respectively. The phenol ring has a C—O bond, O1—C17 and O2—C39, with a bond length of 1.368 (2) and 1.371 (2) Å. The bond angles of the nitrogen and carbon atoms of the central C—N double bond were 118.53 (15); 119.84 (15)° and 123.23 (16); 123.46 (16)°, which indicate the sp^2^ hybridization of these atoms. The observed bond lengths and angles compare well with those found in similar compounds (Ünver *et al.*, 2008; De *et al.*, 2008). The angles between the planes of the anthracene and phenyl rings, C16—N1—C15—C1 and C38—N2—C37—C23, are 174.06 (15) and 179.85 (14)°, respectively.

### Experimental

Synthetic procedures were carried out using standard techniques. Solvents and reagents were used as received. The melting point was determined in open capillaries and is uncorrected. ^1^H and ^13^C NMR spectra were recorded on a JEOL ECX 300 MHz spectrometer using TMS as the internal standard. The IR spectrum was recorded as a KBr disk on a JASCO 460 FTIR. Mass spectrometry was provided by the Washington University Mass Spectrometry Resource with support from the NIH National Center for Research Resources (Grant No. P41RR0954).

The title compound was synthesized using a modification of the method of De *et al.* (2008). 20 ml of methanol, 9-anthracenecarboxaldehyde (0.251 g, 1.22 mmol), and 2-amino-*p*-cresol (0.124 g, 1.01 mmol), and four drops of acetic acid were added to a 50 ml round bottom flask with a magnetic stir bar. The solution was refluxed for 1.5 hours until it was a bright orange color. The solution was then gravity filtered hot and allowed to slowly cool, yielding 0.185 g (59% yield) of bright orange-yellow needle-like crystals.

MP 170-174° C; IR (KBr disk) 3465, 3356, 3052, 3018, 2922, 2860, 1604, 1502 cm^–1^; ^1^H NMR (300 MHz, CDCl_3_) ppm 9.84 (s, 1H), 8.69 (d, 2H), 8.51 (s, 1H), 8.02 (d, 2H), 7.54 (m, 5H), 7.31 (s, 1H), 7.11 (m, 1H), 7.02 (d, 1H), 2.43 (s, 3H); ^13^C NMR (75 MHz, CDCl_3_) ppm 157.8, 150.0, 137.9, 131.5, 130.6, 130.5, 129.1, 129.0, 128.9, 128.1, 127.2, 125.6, 125.2, 118.3, 115.4, 21.1; EI—HR—MS: m/z for [M+H]^+^ = 312.1373, Calcd. m/z for [M+H]^+^ = 312.1388.

### Refinement

Hydrogen atoms on C were placed in idealized positions with C—H bond distances 0.95 - 0.98 Å and thereafter treated as riding. Displacement parameters for H were assigned as U_iso_ = 1.2U_eq_ of the attached atom (1.5 for methyl and OH). A torsional parameter was refined for each methyl group, and OH hydrogen positions were refined.

### Crystal data

**Table:**

C_22_H_17_NO	*Z* = 4
*M**_r_* = 311.37	*F*(000) = 656
Triclinic, *P*1	*D*_x_ = 1.320 Mg m^−^^3^
Hall symbol: -P 1	Mo *K*α radiation, λ = 0.71073 Å
*a* = 8.6037 (15) Å	Cell parameters from 7377 reflections
*b* = 12.839 (3) Å	θ = 2.5–27.8°
*c* = 15.015 (3) Å	µ = 0.08 mm^−^^1^
α = 94.508 (9)°	*T* = 90 K
β = 97.164 (11)°	Lath, yellow
γ = 106.490 (11)°	0.37 × 0.15 × 0.05 mm
*V* = 1566.6 (6) Å^3^	

### Data collection

**Table:**

Nonius KappaCCD diffractometer with Oxford Cryostream	4454 reflections with *I* > 2σ(*I*)
Radiation source: fine-focus sealed tube	*R*_int_ = 0.057
graphite	θ_max_ = 27.9°, θ_min_ = 2.5°
ω scans with κ offsets	*h* = −11→11
35942 measured reflections	*k* = −16→16
7462 independent reflections	*l* = −19→19

### Refinement

**Table:**

Refinement on *F*^2^	Secondary atom site location: difference Fourier map
Least-squares matrix: full	Hydrogen site location: inferred from neighbouring sites
*R*[*F*^2^ > 2σ(*F*^2^)] = 0.054	H atoms treated by a mixture of independent and constrained refinement
*wR*(*F*^2^) = 0.127	*w* = 1/[σ^2^(*F*_o_^2^) + (0.0571*P*)^2^] where *P* = (*F*_o_^2^ + 2*F*_c_^2^)/3
*S* = 1.01	(Δ/σ)_max_ < 0.001
7462 reflections	Δρ_max_ = 0.30 e Å^−^^3^
442 parameters	Δρ_min_ = −0.28 e Å^−^^3^
0 restraints	Extinction correction: *SHELXL97* (Sheldrick, 2008), Fc^*^=kFc[1+0.001xFc^2^λ^3^/sin(2θ)]^-1/4^
Primary atom site location: structure-invariant direct methods	Extinction coefficient: 0.0034 (10)

### Special details

**Table:**

Geometry. All esds (except the esd in the dihedral angle between two l.s. planes) are estimated using the full covariance matrix. The cell esds are taken into account individually in the estimation of esds in distances, angles and torsion angles; correlations between esds in cell parameters are only used when they are defined by crystal symmetry. An approximate (isotropic) treatment of cell esds is used for estimating esds involving l.s. planes.
Refinement. Refinement of *F*^2^ against ALL reflections. The weighted *R*-factor wR and goodness of fit *S* are based on *F*^2^, conventional *R*-factors *R* are based on *F*, with *F* set to zero for negative *F*^2^. The threshold expression of *F*^2^ > σ(*F*^2^) is used only for calculating *R*-factors(gt) etc. and is not relevant to the choice of reflections for refinement. *R*-factors based on *F*^2^ are statistically about twice as large as those based on *F*, and *R*- factors based on ALL data will be even larger.

### Fractional atomic coordinates and isotropic or equivalent isotropic displacement parameters (Å^2^)

**Table:**

	*x*	*y*	*z*	*U*_iso_*/*U*_eq_	
O1	0.30490 (15)	0.14730 (11)	0.20434 (9)	0.0223 (3)	
H10H	0.261 (2)	0.1946 (17)	0.2172 (14)	0.033*	
N1	0.13998 (16)	0.26290 (12)	0.10202 (10)	0.0177 (3)	
C1	0.0939 (2)	0.44058 (15)	0.09855 (11)	0.0164 (4)	
C2	−0.0671 (2)	0.42007 (15)	0.11964 (11)	0.0167 (4)	
C3	−0.1820 (2)	0.31351 (16)	0.10892 (12)	0.0215 (4)	
H3	−0.1516	0.2527	0.0851	0.026*	
C4	−0.3344 (2)	0.29700 (16)	0.13208 (13)	0.0246 (5)	
H4	−0.4084	0.2251	0.1244	0.029*	
C5	−0.3837 (2)	0.38605 (17)	0.16758 (13)	0.0249 (5)	
H5	−0.4894	0.3733	0.1850	0.030*	
C6	−0.2806 (2)	0.48933 (16)	0.17677 (12)	0.0213 (4)	
H6	−0.3157	0.5486	0.1994	0.026*	
C7	−0.1195 (2)	0.51040 (15)	0.15290 (11)	0.0170 (4)	
C8	−0.0120 (2)	0.61631 (15)	0.16377 (12)	0.0196 (4)	
H8	−0.0473	0.6754	0.1867	0.023*	
C9	0.1462 (2)	0.63784 (15)	0.14184 (12)	0.0181 (4)	
C10	0.2576 (2)	0.74645 (16)	0.15396 (13)	0.0237 (4)	
H10	0.2225	0.8059	0.1763	0.028*	
C11	0.4120 (2)	0.76605 (17)	0.13417 (14)	0.0289 (5)	
H11	0.4841	0.8386	0.1425	0.035*	
C12	0.4657 (2)	0.67730 (16)	0.10083 (13)	0.0262 (5)	
H12	0.5740	0.6911	0.0870	0.031*	
C13	0.3642 (2)	0.57331 (16)	0.08864 (13)	0.0222 (4)	
H13	0.4031	0.5156	0.0665	0.027*	
C14	0.2004 (2)	0.54824 (15)	0.10823 (12)	0.0178 (4)	
C15	0.1512 (2)	0.34955 (15)	0.06342 (12)	0.0181 (4)	
H15	0.1987	0.3556	0.0095	0.022*	
C16	0.1843 (2)	0.17612 (14)	0.05799 (12)	0.0165 (4)	
C17	0.2645 (2)	0.11812 (15)	0.11268 (12)	0.0170 (4)	
C18	0.3106 (2)	0.03190 (15)	0.07482 (12)	0.0191 (4)	
H18	0.3682	−0.0058	0.1120	0.023*	
C19	0.2723 (2)	0.00072 (15)	−0.01782 (13)	0.0218 (4)	
H19	0.3061	−0.0577	−0.0435	0.026*	
C20	0.1853 (2)	0.05329 (15)	−0.07398 (12)	0.0202 (4)	
C21	0.1432 (2)	0.14129 (16)	−0.03464 (12)	0.0198 (4)	
H21	0.0850	0.1787	−0.0718	0.024*	
C22	0.1364 (2)	0.01475 (18)	−0.17348 (13)	0.0316 (5)	
H22A	0.0264	−0.0380	−0.1843	0.047*	
H22B	0.2149	−0.0203	−0.1943	0.047*	
H22C	0.1359	0.0774	−0.2067	0.047*	
O2	0.53743 (15)	0.04269 (11)	0.27603 (8)	0.0207 (3)	
H20H	0.488 (2)	0.0923 (17)	0.2760 (13)	0.031*	
N2	0.52578 (16)	0.20557 (12)	0.39729 (10)	0.0168 (3)	
C23	0.48926 (19)	0.38573 (14)	0.40197 (11)	0.0142 (4)	
C24	0.3154 (2)	0.35684 (14)	0.38026 (11)	0.0150 (4)	
C25	0.2085 (2)	0.25083 (15)	0.38727 (11)	0.0175 (4)	
H25	0.2540	0.1949	0.4049	0.021*	
C26	0.0420 (2)	0.22873 (15)	0.36905 (12)	0.0197 (4)	
H26	−0.0263	0.1578	0.3746	0.024*	
C27	−0.0304 (2)	0.30931 (16)	0.34210 (12)	0.0205 (4)	
H27	−0.1463	0.2922	0.3290	0.025*	
C28	0.0661 (2)	0.41141 (16)	0.33494 (12)	0.0195 (4)	
H28	0.0167	0.4656	0.3175	0.023*	
C29	0.2409 (2)	0.43844 (15)	0.35330 (11)	0.0161 (4)	
C30	0.3395 (2)	0.54295 (15)	0.34506 (11)	0.0172 (4)	
H30	0.2891	0.5956	0.3249	0.021*	
C31	0.5098 (2)	0.57245 (14)	0.36561 (11)	0.0167 (4)	
C32	0.6089 (2)	0.67977 (15)	0.35648 (13)	0.0229 (4)	
H32	0.5579	0.7320	0.3359	0.027*	
C33	0.7750 (2)	0.70842 (15)	0.37681 (13)	0.0236 (4)	
H33	0.8392	0.7804	0.3709	0.028*	
C34	0.8524 (2)	0.63117 (15)	0.40679 (12)	0.0211 (4)	
H34	0.9686	0.6519	0.4210	0.025*	
C35	0.7631 (2)	0.52775 (15)	0.41557 (12)	0.0188 (4)	
H35	0.8181	0.4771	0.4350	0.023*	
C36	0.5874 (2)	0.49345 (14)	0.39604 (11)	0.0152 (4)	
C37	0.5733 (2)	0.30621 (15)	0.43076 (12)	0.0157 (4)	
H37	0.6671	0.3307	0.4763	0.019*	
C38	0.61304 (19)	0.13383 (14)	0.42820 (12)	0.0145 (4)	
C39	0.61383 (19)	0.05028 (15)	0.36290 (12)	0.0157 (4)	
C40	0.69549 (19)	−0.02469 (14)	0.38538 (12)	0.0164 (4)	
H40	0.6983	−0.0807	0.3409	0.020*	
C41	0.77342 (19)	−0.01782 (15)	0.47339 (12)	0.0172 (4)	
H41	0.8307	−0.0690	0.4881	0.021*	
C42	0.76957 (19)	0.06220 (15)	0.54060 (12)	0.0172 (4)	
C43	0.68877 (19)	0.13757 (14)	0.51648 (12)	0.0161 (4)	
H43	0.6850	0.1930	0.5612	0.019*	
C44	0.8441 (2)	0.06293 (16)	0.63718 (12)	0.0219 (4)	
H44A	0.8793	0.1379	0.6678	0.033*	
H44B	0.9391	0.0350	0.6380	0.033*	
H44C	0.7624	0.0163	0.6685	0.033*	

### Atomic displacement parameters (Å^2^)

**Table:**

	*U*^11^	*U*^22^	*U*^33^	*U*^12^	*U*^13^	*U*^23^
O1	0.0282 (7)	0.0235 (8)	0.0179 (7)	0.0142 (6)	−0.0002 (6)	0.0010 (6)
N1	0.0183 (8)	0.0189 (9)	0.0177 (8)	0.0092 (6)	0.0015 (6)	0.0003 (7)
C1	0.0197 (9)	0.0186 (10)	0.0134 (9)	0.0097 (8)	0.0013 (7)	0.0030 (8)
C2	0.0191 (9)	0.0201 (10)	0.0128 (9)	0.0099 (8)	−0.0006 (7)	0.0029 (8)
C3	0.0212 (10)	0.0212 (11)	0.0224 (10)	0.0094 (8)	0.0001 (8)	−0.0016 (8)
C4	0.0196 (9)	0.0238 (11)	0.0290 (11)	0.0055 (8)	0.0021 (8)	0.0014 (9)
C5	0.0202 (10)	0.0313 (12)	0.0253 (11)	0.0110 (9)	0.0043 (8)	0.0018 (9)
C6	0.0217 (10)	0.0283 (12)	0.0184 (10)	0.0151 (9)	0.0032 (8)	0.0009 (8)
C7	0.0195 (9)	0.0232 (11)	0.0116 (9)	0.0121 (8)	0.0008 (7)	0.0036 (8)
C8	0.0262 (10)	0.0200 (11)	0.0173 (10)	0.0150 (8)	0.0018 (8)	0.0028 (8)
C9	0.0221 (9)	0.0204 (11)	0.0139 (9)	0.0098 (8)	0.0010 (8)	0.0042 (8)
C10	0.0311 (11)	0.0165 (10)	0.0264 (11)	0.0118 (8)	0.0034 (9)	0.0034 (9)
C11	0.0289 (11)	0.0217 (12)	0.0369 (13)	0.0070 (9)	0.0053 (9)	0.0087 (10)
C12	0.0225 (10)	0.0264 (12)	0.0333 (12)	0.0099 (9)	0.0077 (9)	0.0095 (10)
C13	0.0240 (10)	0.0228 (11)	0.0248 (11)	0.0136 (8)	0.0051 (8)	0.0062 (9)
C14	0.0217 (9)	0.0201 (11)	0.0138 (9)	0.0103 (8)	0.0006 (7)	0.0038 (8)
C15	0.0162 (9)	0.0228 (11)	0.0162 (10)	0.0077 (8)	0.0013 (7)	0.0021 (8)
C16	0.0151 (8)	0.0163 (10)	0.0194 (10)	0.0056 (7)	0.0054 (7)	0.0016 (8)
C17	0.0163 (9)	0.0159 (10)	0.0176 (10)	0.0033 (7)	0.0029 (8)	0.0000 (8)
C18	0.0176 (9)	0.0169 (10)	0.0232 (11)	0.0067 (8)	0.0002 (8)	0.0030 (8)
C19	0.0168 (9)	0.0189 (11)	0.0287 (11)	0.0045 (8)	0.0057 (8)	−0.0043 (9)
C20	0.0148 (9)	0.0242 (11)	0.0191 (10)	0.0030 (8)	0.0034 (8)	−0.0034 (8)
C21	0.0166 (9)	0.0244 (11)	0.0188 (10)	0.0060 (8)	0.0027 (8)	0.0052 (8)
C22	0.0275 (11)	0.0411 (14)	0.0234 (11)	0.0081 (10)	0.0041 (9)	−0.0059 (10)
O2	0.0236 (7)	0.0198 (8)	0.0208 (7)	0.0118 (6)	−0.0003 (6)	0.0000 (6)
N2	0.0180 (8)	0.0160 (9)	0.0193 (8)	0.0086 (6)	0.0051 (6)	0.0032 (7)
C23	0.0185 (9)	0.0141 (10)	0.0117 (9)	0.0076 (7)	0.0028 (7)	0.0001 (7)
C24	0.0202 (9)	0.0162 (10)	0.0101 (9)	0.0083 (7)	0.0025 (7)	−0.0003 (7)
C25	0.0201 (9)	0.0177 (10)	0.0179 (10)	0.0092 (8)	0.0052 (8)	0.0036 (8)
C26	0.0195 (9)	0.0170 (10)	0.0214 (10)	0.0032 (8)	0.0048 (8)	0.0000 (8)
C27	0.0148 (9)	0.0281 (12)	0.0193 (10)	0.0085 (8)	0.0007 (8)	0.0017 (8)
C28	0.0213 (9)	0.0255 (11)	0.0157 (10)	0.0142 (8)	0.0006 (8)	0.0021 (8)
C29	0.0210 (9)	0.0204 (10)	0.0095 (9)	0.0107 (8)	0.0021 (7)	0.0003 (8)
C30	0.0250 (10)	0.0163 (10)	0.0150 (9)	0.0127 (8)	0.0043 (8)	0.0036 (8)
C31	0.0243 (9)	0.0143 (10)	0.0139 (9)	0.0079 (8)	0.0060 (8)	0.0017 (8)
C32	0.0314 (11)	0.0156 (10)	0.0249 (11)	0.0096 (8)	0.0087 (9)	0.0049 (8)
C33	0.0300 (11)	0.0115 (10)	0.0286 (11)	0.0031 (8)	0.0100 (9)	0.0006 (8)
C34	0.0199 (9)	0.0191 (11)	0.0218 (10)	0.0027 (8)	0.0053 (8)	−0.0039 (8)
C35	0.0209 (9)	0.0195 (10)	0.0176 (10)	0.0084 (8)	0.0048 (8)	0.0005 (8)
C36	0.0208 (9)	0.0131 (10)	0.0120 (9)	0.0056 (7)	0.0042 (7)	−0.0006 (7)
C37	0.0154 (9)	0.0185 (10)	0.0145 (9)	0.0067 (7)	0.0028 (7)	0.0021 (8)
C38	0.0128 (8)	0.0126 (9)	0.0201 (10)	0.0048 (7)	0.0060 (7)	0.0049 (8)
C39	0.0124 (8)	0.0190 (10)	0.0149 (9)	0.0028 (7)	0.0023 (7)	0.0036 (8)
C40	0.0161 (9)	0.0135 (10)	0.0216 (10)	0.0058 (7)	0.0071 (8)	0.0023 (8)
C41	0.0136 (8)	0.0162 (10)	0.0254 (10)	0.0064 (7)	0.0071 (8)	0.0089 (8)
C42	0.0127 (8)	0.0193 (10)	0.0209 (10)	0.0039 (7)	0.0059 (7)	0.0076 (8)
C43	0.0157 (9)	0.0134 (10)	0.0190 (10)	0.0037 (7)	0.0043 (7)	0.0007 (8)
C44	0.0210 (9)	0.0244 (11)	0.0224 (11)	0.0087 (8)	0.0040 (8)	0.0069 (9)

### Geometric parameters (Å, °)

**Table:**

O1—C17	1.371 (2)	O2—C39	1.368 (2)
O1—H10H	0.82 (2)	O2—H20H	0.86 (2)
N1—C15	1.280 (2)	N2—C37	1.279 (2)
N1—C16	1.419 (2)	N2—C38	1.414 (2)
C1—C14	1.410 (2)	C23—C36	1.417 (2)
C1—C2	1.416 (2)	C23—C24	1.423 (2)
C1—C15	1.477 (2)	C23—C37	1.470 (2)
C2—C3	1.426 (2)	C24—C25	1.430 (2)
C2—C7	1.435 (2)	C24—C29	1.434 (2)
C3—C4	1.361 (2)	C25—C26	1.366 (2)
C3—H3	0.9500	C25—H25	0.9500
C4—C5	1.417 (3)	C26—C27	1.411 (3)
C4—H4	0.9500	C26—H26	0.9500
C5—C6	1.356 (3)	C27—C28	1.359 (3)
C5—H5	0.9500	C27—H27	0.9500
C6—C7	1.431 (2)	C28—C29	1.429 (2)
C6—H6	0.9500	C28—H28	0.9500
C7—C8	1.395 (2)	C29—C30	1.393 (2)
C8—C9	1.396 (2)	C30—C31	1.393 (2)
C8—H8	0.9500	C30—H30	0.9500
C9—C10	1.433 (3)	C31—C32	1.427 (2)
C9—C14	1.438 (2)	C31—C36	1.437 (2)
C10—C11	1.355 (3)	C32—C33	1.359 (3)
C10—H10	0.9500	C32—H32	0.9500
C11—C12	1.425 (3)	C33—C34	1.414 (3)
C11—H11	0.9500	C33—H33	0.9500
C12—C13	1.356 (3)	C34—C35	1.358 (2)
C12—H12	0.9500	C34—H34	0.9500
C13—C14	1.427 (2)	C35—C36	1.434 (2)
C13—H13	0.9500	C35—H35	0.9500
C15—H15	0.9500	C37—H37	0.9500
C16—C21	1.396 (2)	C38—C43	1.393 (2)
C16—C17	1.397 (2)	C38—C39	1.397 (2)
C17—C18	1.383 (2)	C39—C40	1.382 (2)
C18—C19	1.388 (3)	C40—C41	1.389 (2)
C18—H18	0.9500	C40—H40	0.9500
C19—C20	1.395 (3)	C41—C42	1.393 (2)
C19—H19	0.9500	C41—H41	0.9500
C20—C21	1.392 (2)	C42—C43	1.388 (2)
C20—C22	1.504 (3)	C42—C44	1.509 (2)
C21—H21	0.9500	C43—H43	0.9500
C22—H22A	0.9800	C44—H44A	0.9800
C22—H22B	0.9800	C44—H44B	0.9800
C22—H22C	0.9800	C44—H44C	0.9800
			
C17—O1—H10H	106.8 (15)	C39—O2—H20H	105.9 (14)
C15—N1—C16	118.53 (15)	C37—N2—C38	119.84 (15)
C14—C1—C2	120.64 (16)	C36—C23—C24	120.39 (16)
C14—C1—C15	118.97 (15)	C36—C23—C37	117.76 (15)
C2—C1—C15	120.36 (16)	C24—C23—C37	121.85 (16)
C1—C2—C3	123.32 (16)	C23—C24—C25	123.46 (16)
C1—C2—C7	119.01 (16)	C23—C24—C29	119.12 (16)
C3—C2—C7	117.67 (15)	C25—C24—C29	117.37 (15)
C4—C3—C2	121.46 (18)	C26—C25—C24	121.06 (17)
C4—C3—H3	119.3	C26—C25—H25	119.5
C2—C3—H3	119.3	C24—C25—H25	119.5
C3—C4—C5	120.64 (18)	C25—C26—C27	121.23 (17)
C3—C4—H4	119.7	C25—C26—H26	119.4
C5—C4—H4	119.7	C27—C26—H26	119.4
C6—C5—C4	120.15 (17)	C28—C27—C26	119.87 (16)
C6—C5—H5	119.9	C28—C27—H27	120.1
C4—C5—H5	119.9	C26—C27—H27	120.1
C5—C6—C7	121.02 (18)	C27—C28—C29	120.93 (17)
C5—C6—H6	119.5	C27—C28—H28	119.5
C7—C6—H6	119.5	C29—C28—H28	119.5
C8—C7—C6	121.19 (17)	C30—C29—C28	120.78 (16)
C8—C7—C2	119.78 (15)	C30—C29—C24	119.70 (15)
C6—C7—C2	119.00 (16)	C28—C29—C24	119.53 (16)
C7—C8—C9	121.78 (17)	C31—C30—C29	121.87 (16)
C7—C8—H8	119.1	C31—C30—H30	119.1
C9—C8—H8	119.1	C29—C30—H30	119.1
C8—C9—C10	121.89 (17)	C30—C31—C32	121.12 (17)
C8—C9—C14	119.11 (16)	C30—C31—C36	119.56 (16)
C10—C9—C14	118.98 (16)	C32—C31—C36	119.32 (16)
C11—C10—C9	121.35 (18)	C33—C32—C31	121.00 (18)
C11—C10—H10	119.3	C33—C32—H32	119.5
C9—C10—H10	119.3	C31—C32—H32	119.5
C10—C11—C12	119.68 (18)	C32—C33—C34	120.05 (17)
C10—C11—H11	120.2	C32—C33—H33	120.0
C12—C11—H11	120.2	C34—C33—H33	120.0
C13—C12—C11	120.79 (17)	C35—C34—C33	120.98 (17)
C13—C12—H12	119.6	C35—C34—H34	119.5
C11—C12—H12	119.6	C33—C34—H34	119.5
C12—C13—C14	121.78 (18)	C34—C35—C36	121.33 (17)
C12—C13—H13	119.1	C34—C35—H35	119.3
C14—C13—H13	119.1	C36—C35—H35	119.3
C1—C14—C13	122.90 (17)	C23—C36—C35	123.36 (16)
C1—C14—C9	119.66 (15)	C23—C36—C31	119.28 (15)
C13—C14—C9	117.42 (16)	C35—C36—C31	117.32 (16)
N1—C15—C1	123.23 (16)	N2—C37—C23	122.99 (16)
N1—C15—H15	118.4	N2—C37—H37	118.5
C1—C15—H15	118.4	C23—C37—H37	118.5
C21—C16—C17	118.71 (16)	C43—C38—C39	119.36 (16)
C21—C16—N1	124.14 (16)	C43—C38—N2	125.80 (16)
C17—C16—N1	117.01 (15)	C39—C38—N2	114.77 (16)
O1—C17—C18	118.31 (16)	O2—C39—C40	119.20 (16)
O1—C17—C16	121.24 (16)	O2—C39—C38	120.91 (16)
C18—C17—C16	120.41 (16)	C40—C39—C38	119.88 (16)
C17—C18—C19	119.72 (17)	C39—C40—C41	119.70 (17)
C17—C18—H18	120.1	C39—C40—H40	120.2
C19—C18—H18	120.1	C41—C40—H40	120.2
C18—C19—C20	121.45 (17)	C40—C41—C42	121.63 (17)
C18—C19—H19	119.3	C40—C41—H41	119.2
C20—C19—H19	119.3	C42—C41—H41	119.2
C21—C20—C19	117.80 (17)	C43—C42—C41	117.83 (17)
C21—C20—C22	121.23 (18)	C43—C42—C44	121.43 (17)
C19—C20—C22	120.97 (17)	C41—C42—C44	120.68 (16)
C20—C21—C16	121.79 (17)	C42—C43—C38	121.54 (17)
C20—C21—H21	119.1	C42—C43—H43	119.2
C16—C21—H21	119.1	C38—C43—H43	119.2
C20—C22—H22A	109.5	C42—C44—H44A	109.5
C20—C22—H22B	109.5	C42—C44—H44B	109.5
H22A—C22—H22B	109.5	H44A—C44—H44B	109.5
C20—C22—H22C	109.5	C42—C44—H44C	109.5
H22A—C22—H22C	109.5	H44A—C44—H44C	109.5
H22B—C22—H22C	109.5	H44B—C44—H44C	109.5
			
C14—C1—C2—C3	178.20 (16)	C36—C23—C24—C25	−178.08 (15)
C15—C1—C2—C3	0.0 (3)	C37—C23—C24—C25	2.1 (3)
C14—C1—C2—C7	−1.3 (3)	C36—C23—C24—C29	−0.7 (2)
C15—C1—C2—C7	−179.45 (16)	C37—C23—C24—C29	179.52 (16)
C1—C2—C3—C4	178.37 (17)	C23—C24—C25—C26	177.33 (16)
C7—C2—C3—C4	−2.1 (3)	C29—C24—C25—C26	−0.1 (2)
C2—C3—C4—C5	0.2 (3)	C24—C25—C26—C27	0.5 (3)
C3—C4—C5—C6	1.6 (3)	C25—C26—C27—C28	−0.8 (3)
C4—C5—C6—C7	−1.4 (3)	C26—C27—C28—C29	0.8 (3)
C5—C6—C7—C8	−178.84 (17)	C27—C28—C29—C30	179.40 (17)
C5—C6—C7—C2	−0.6 (3)	C27—C28—C29—C24	−0.4 (2)
C1—C2—C7—C8	0.1 (2)	C23—C24—C29—C30	2.7 (2)
C3—C2—C7—C8	−179.44 (16)	C25—C24—C29—C30	−179.73 (16)
C1—C2—C7—C6	−178.15 (16)	C23—C24—C29—C28	−177.46 (15)
C3—C2—C7—C6	2.3 (2)	C25—C24—C29—C28	0.1 (2)
C6—C7—C8—C9	179.17 (17)	C28—C29—C30—C31	177.71 (15)
C2—C7—C8—C9	1.0 (3)	C24—C29—C30—C31	−2.5 (3)
C7—C8—C9—C10	−179.25 (17)	C29—C30—C31—C32	179.78 (16)
C7—C8—C9—C14	−0.8 (3)	C29—C30—C31—C36	0.1 (3)
C8—C9—C10—C11	178.62 (18)	C30—C31—C32—C33	179.82 (17)
C14—C9—C10—C11	0.2 (3)	C36—C31—C32—C33	−0.5 (3)
C9—C10—C11—C12	−0.2 (3)	C31—C32—C33—C34	0.5 (3)
C10—C11—C12—C13	0.0 (3)	C32—C33—C34—C35	0.2 (3)
C11—C12—C13—C14	0.2 (3)	C33—C34—C35—C36	−0.9 (3)
C2—C1—C14—C13	179.46 (16)	C24—C23—C36—C35	−179.05 (15)
C15—C1—C14—C13	−2.3 (3)	C37—C23—C36—C35	0.8 (2)
C2—C1—C14—C9	1.5 (3)	C24—C23—C36—C31	−1.6 (2)
C15—C1—C14—C9	179.64 (16)	C37—C23—C36—C31	178.21 (16)
C12—C13—C14—C1	−178.18 (17)	C34—C35—C36—C23	178.33 (16)
C12—C13—C14—C9	−0.1 (3)	C34—C35—C36—C31	0.8 (2)
C8—C9—C14—C1	−0.4 (3)	C30—C31—C36—C23	1.9 (2)
C10—C9—C14—C1	178.07 (16)	C32—C31—C36—C23	−177.74 (15)
C8—C9—C14—C13	−178.52 (17)	C30—C31—C36—C35	179.50 (15)
C10—C9—C14—C13	0.0 (2)	C32—C31—C36—C35	−0.1 (2)
C16—N1—C15—C1	174.06 (15)	C38—N2—C37—C23	179.85 (14)
C14—C1—C15—N1	130.13 (19)	C36—C23—C37—N2	−142.22 (16)
C2—C1—C15—N1	−51.7 (3)	C24—C23—C37—N2	37.6 (2)
C15—N1—C16—C21	−41.7 (2)	C37—N2—C38—C43	35.3 (2)
C15—N1—C16—C17	142.73 (16)	C37—N2—C38—C39	−147.70 (15)
C21—C16—C17—O1	−178.70 (15)	C43—C38—C39—O2	178.51 (14)
N1—C16—C17—O1	−2.9 (2)	N2—C38—C39—O2	1.3 (2)
C21—C16—C17—C18	3.7 (2)	C43—C38—C39—C40	−2.8 (2)
N1—C16—C17—C18	179.53 (15)	N2—C38—C39—C40	179.93 (14)
O1—C17—C18—C19	−179.66 (15)	O2—C39—C40—C41	−179.96 (14)
C16—C17—C18—C19	−2.0 (2)	C38—C39—C40—C41	1.4 (2)
C17—C18—C19—C20	−1.2 (3)	C39—C40—C41—C42	1.0 (2)
C18—C19—C20—C21	2.4 (2)	C40—C41—C42—C43	−1.8 (2)
C18—C19—C20—C22	−176.54 (16)	C40—C41—C42—C44	175.37 (15)
C19—C20—C21—C16	−0.6 (2)	C41—C42—C43—C38	0.2 (2)
C22—C20—C21—C16	178.34 (16)	C44—C42—C43—C38	−176.88 (15)
C17—C16—C21—C20	−2.4 (2)	C39—C38—C43—C42	2.0 (2)
N1—C16—C21—C20	−177.88 (15)	N2—C38—C43—C42	178.95 (15)

### Hydrogen-bond geometry (Å, °)

**Table:**

*D*—H···*A*	*D*—H	H···*A*	*D*···*A*	*D*—H···*A*
O1—H10H···N1	0.82 (2)	2.27 (2)	2.754 (2)	118.0 (17)
O2—H20H···O1	0.86 (2)	2.11 (2)	2.8602 (18)	144.9 (18)
O2—H20H···N2	0.86 (2)	2.17 (2)	2.695 (2)	119.1 (17)

## References

[bb1] Allen, F. H., Kennard, O., Watson, D. G., Brammmer, L., Orpen, A. G. & Taylor, R. (1987). *J. Chem. Soc. Perkin Trans. 2*, pp. S1–19.

[bb2] Altomare, A., Burla, M. C., Camalli, M., Cascarano, G. L., Giacovazzo, C., Guagliardi, A., Moliterni, A. G. G., Polidori, G. & Spagna, R. (1999). *J. Appl. Cryst.* **32**, 115–119.

[bb3] De, R. L., Mandal, M., Roy, L. & Mukherjee, J. (2008). *Indian J. Chem. Sect. A*, **47**, 207–213.

[bb4] Farrugia, L. J. (1997). *J. Appl. Cryst.* **30**, 565.

[bb5] Liao, S.-H., Shiu, J.-R., Liu, S.-W., Yeh, S.-J., Chen, Y.-H., Chen, C.-T., Chow, T. J. & Wu, C.-I. (2009). *J. Am. Chem. Soc.* **131**, 763–777. 10.1021/ja807284e 19093863

[bb6] Mak, C. S. K., Wong, H. L., Leung, Q. Y., Tam, W. Y., Chan, W. K. & Djurišić, A. B. (2009). *J. Organomet. Chem.* **694**, 2770–2776.

[bb7] Nonius (2000). *COLLECT* Nonius BV, Delft, The Netherlands.

[bb8] Otwinowski, Z. & Minor, W. (1997). *Methods in Enzymology*, Vol. 276, *Macromolecular Crystallography*, Part A, edited by C. W. Carter Jr & R. M. Sweet, pp. 307–326. New York: Academic Press.

[bb9] Sheldrick, G. M. (2008). *Acta Cryst.* A**64**, 112–122. 10.1107/S0108767307043930 18156677

[bb10] Ünver, H., Yildiz, M., Kiraz, A. & Özgen, Ö. (2009). *J. Chem. Crystallogr.* **39**, 17–23.

